# A Dual-Functional and Efficient MOF-5@MWCNTs Electrochemical Sensing Device for the Measurement of Trace-Level Acetaminophenol and Dopamine

**DOI:** 10.3390/molecules29235534

**Published:** 2024-11-23

**Authors:** Jianxia Gu, Shuting Lang, Zhanbin Jin, Tingting Wei

**Affiliations:** Department of Chemistry, Xinzhou Normal University, Xinzhou 034000, China; 221124010518@zjut.edu.cn (S.L.); jinzb959@nenu.edu.cn (Z.J.); weitt321@nenu.edu.cn (T.W.)

**Keywords:** multi-walled carbon nanotubes, metal–organic framework, electrochemical sensor, acetaminophen, dopamine, trace determination

## Abstract

The design and construction of dual-functional and high-efficiency electrochemical sensors are necessary for quantitative detection. In this work, a zinc-based metal–organic framework (MOF-5) and multi-walled carbon nanotubes (MWCNTs) were combined in situ through a simple solvothermal reaction to obtain an MOF-5@MWCNTs composite. The composite exhibits a large surface area, hierarchical pore structure, excellent conductivity, and enhanced electrochemical performance in the detection of acetaminophenol (AP) and dopamine (DA). Remarkably, the synergistic effects between MOF-5 and MWCNTs enable the electrochemical sensor based on the MOF-5@MWCNTs composite to quantitatively determine AP and DA at trace levels. Under optimal conditions, the proposed sensor features relatively wide linear ranges of 0.005–600 μM and 0.1–60 μM for AP and DA, respectively, with very low detection limits (LODs) of 0.061 μM and 0.0075 μM for AP and DA. Importantly, this electrochemical sensor demonstrates excellent reproducibility, stability, and anti-interference ability, making it suitable for practical applications in the detection of AP and DA in urine and tap water samples with acceptable recoveries. The successful integration of MOF-5 with MWCNTs results in a robust and versatile electrochemical sensing platform for the rapid and reliable detection of AP and DA at trace levels.

## 1. Introduction

Acetaminophenol (AP, C_8_H_9_NO_2_) is a widely consumed analgesic and antipyretic agent due to its availability and inexpensiveness, and was extensively used during the COVID-19 pandemic [1,2]. However, overdoses or prolonged misuse can lead to severe hepatotoxicity, necessitating a close monitoring of serum levels for timely intervention [3]. Dopamine (DA, 3,4-dihydroxy phenylalanine), which has a similar structure to acetaminophen, is a critical neurotransmitter involved in various neurological functions, playing a central role in regulating mood, motivation, reward, and motor function. The dysregulation of dopamine levels or a dysfunction in its signaling pathways are implicated in a variety of neurological disorders, including Parkinson’s disease and schizophrenia, among others [4,5]. Therefore, the quantitative detection of AP and DA is of paramount importance across various scientific and medical disciplines. The detection of AP and DA can be achieved through various analytical methods, with electrochemical detection emerging as a highly advantageous approach due to its sensitivity, selectivity, simplicity, rapid analysis, cost-effectiveness, portability, and potential for real-time monitoring [6,7,8]. However, the electrochemical response of determinand on the bare electrode is weak, which limits its application to a certain extent. Therefore, it is necessary to seek out high-performance electrode materials to overcome this limitation.

Metal–organic frameworks (MOFs) are a class of porous, crystalline materials that have recently gained significant attention in electrochemical detection due to their remarkable properties such as high surface area, tunable pore size, and adjustable functionality [9]. Nonetheless, many MOFs exhibit poor electrical conductivity, which can limit electron transfer efficiency and make their integration with electrode surfaces difficult [10,11]. This inefficiency and difficulty of integration can further lead to a poor sensitivity, slow response times, and high detection limits, restricting their utility in detecting analytes requiring rapid and sensitive quantification. To overcome these limitations, researchers have pursued strategies such as composite formation with conductive materials (like carbon nanotubes or graphene), which aims to enhance the electrochemical activity of MOFs, thereby unlocking their full potential in the realm of sensitive and selective electrochemical detection [12].

**Figure 1:**
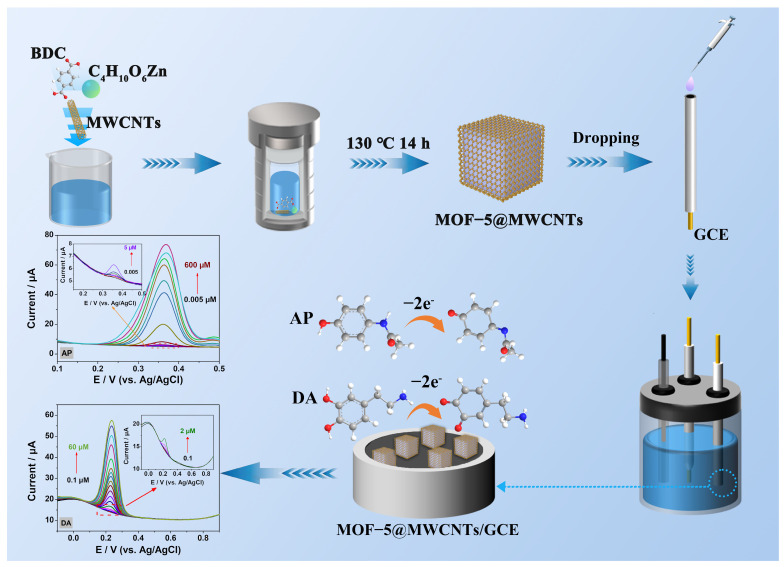
Specific synthesis process for the construction of dual-functional electrochemical sensor based on MOF-5@WMCNTs composite for AP and DA detection.

In this work, a simple solvothermal method was used to successfully compound a zinc-based metal–organic skeleton (MOF-5) with multi-walled carbon nanotubes (MWCNTs) in situ, and a dual-functional electrochemical sensor based on the MOF-5@MWCNTs composite was designed to determine AP and DA with a high efficiency (Figure 1). MOF-5, a prototypical member of the MOF family, has garnered significant attention for its application in electrochemical sensing due to its tunability, stability, scalability, and compatibility with conductive nanomaterials [13]. MWCNTs, known for their exceptional electrical conductivity and large surface area, serve as a conductive platform when interfaced with insulating MOF-5 [14]. This integration facilitates rapid electron transfer, thereby improving the sensitivity and response time of the electrochemical sensor. Additionally, the composite could achieve enhanced mechanical stability by incorporating MWCNTs. As expected, the electrochemical tests show that the sensor based on MOF-5@MWCNTs can perform the quantitative detection of AP or DA well, with a wide linear range and a very low detection limit (LOD). In addition, this sensor also demonstrated an excellent performance in terms of its selectivity, reproducibility, stability, and practicability. This exceptional performance in detection is due to a synergistic combination of properties derived from both components, which collectively overcome limitations inherent to each. The marriage of MWCNTs and MOF-5 in electrochemical detection represents a significant step forward, rendering it a promising platform for advancing the capabilities of electrochemical sensors, particularly in the detection of challenging analytes with high specificities and sensitivities.

## 2. Results and Discussion

### 2.1. Characterization of MOF-5, MWCNTs and MOF-5@MWCNTs

**Figure 2:**
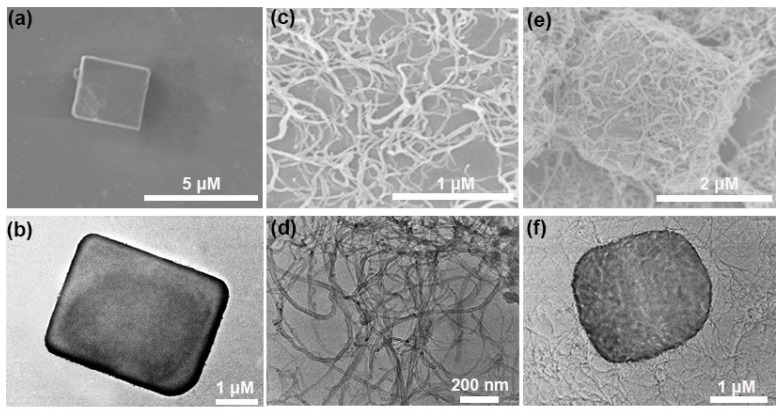
SEM and TEM images of MOF-5 (**a**,**b**), MWCNTs (**c**,**d**), and MOF-5@MWCNTs (**e**,**f**).

The microcosmic morphologies of the MOF-5, MWCNTs, and MOF-5@MWCNTs were characterized through scanning electron microscopy (SEM) and transmission electron microscopy (TEM). Notably, MOF-5 presents a rectangular shape (Figure 2a,b) [15]. MWCNTs exhibit their own characteristic tubular morphology (Figure 2c,d). For the MOF-5@MWCNTs composite, it is clearly seen that the interwoven MWCNTs are draped over MOF-5 like a net, indicating the successful synthesis of the MOF-5@MWCNTs composite (Figure 2e,f).

**Figure 3:**
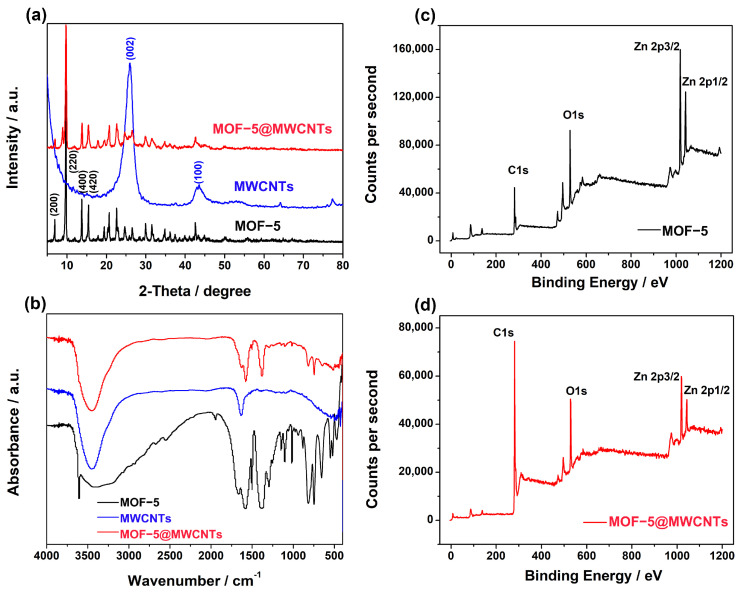
The characterization results of MOF-5, MWCNTs, and MOF-5@MWCNTs: (**a**) XRD patterns, (**b**) FT-IR, (**c**,**d**) XPS spectra.

Powder X-ray diffraction (PXRD) was carried out to determine the structural characteristics of the prepared electrode materials. The XRD patterns of MWCNTs show two characteristic peaks at around 25.96° and 43.42°, which pertain to the typical structure (002) and (100) facets of the MWCNTs, respectively (Figure 3a) [16]. For MOF-5, the clear diffraction peaks at 2θ = 6.90°, 9.74°, 13.84°, and 15.42° are assigned to the (200), (220), (400), and (420) planes of the crystallographic structure of MOF-5, respectively (Figure 3a) [17,18]. Moreover, it is worth noting that the XRD patterns of the MOF-5@MWCNTs composite show characteristic peaks of both MOF-5 and MWCNTs (Figure 3a), indicating that the composite material was prepared successfully and that the MOF-5 in the composite retains satisfactory crystallinity even after the introduction of MWCNTs. MOF-5, MWCNTs, and MOF-5@MWCNTs were characterized by Fourier transform infrared spectroscopy (FT-IR) to investigate their characteristic functional groups, as shown in Figure 3b. For MOF-5, there is a strong peak at 1577 cm^−1^, which is attributed to the stretching vibrations of carboxylate anions. Furthermore, there are no strong absorption bands at 1760–1690 cm^−1^, indicating that the -COOH groups in 1,4-benzenedicarboxylic acid have been deprotonated and reacted with metal ions. Additionally, the broad bands at 3500–3100 cm^−1^ indicate the presence of water molecules within the framework [19]. The pore characteristics and surface area of MWCNTs, MOF-5, MWCNTs, and MOF-5@MWCNTs were investigated by N_2_ adsorption–desorption isotherms (Figure S1) [20]. Evidently, the introduction of MWCNTs makes MOF-5@MWCNTs possess a larger surface area and hierarchical pore structure compared with MOF-5, which would be favorable of mass adsorption and transfer. The elemental composition of pristine MOF-5 and MOF-5@MWCNTs was investigated by X-ray photoelectron spectroscopy (XPS). It is clearly seen that there are C, O, and Zn elements in MOF-5 from a full scan spectrum (Figure 3c) and the binding energies centered at 283.9 eV, 526.07 eV, 1016.42 eV, and 1039.65 eV correspond to C1s, O1s, Zn2p3/2, and Zn2p1/2, respectively, which are consistent with previous reports [21]. Similarly, the peaks of C, O, and Zn elements are also observed in the XPS survey of MOF-5@MWCNTs (Figure 3d). It is worth noting that the peak intensity of C1s significantly increases compared to MOF-5, which is due to the introduction of MWCNTs, confirming the successful synthesis of MOF-5@MWCNTs. 

### 2.2. Characterization of Electrochemical Properties of MOF-5, MWCNTs and MOF-5@MWCNTs

In 0.1 M KCl solution containing 5.0 mM Fe(CN)_6_^3−/4−^, the electron transfer behavior of different electrode materials was investigated by electrochemical impedance spectroscopy (EIS). The charge transfer resistance (R_ct_) of the electrode materials was assessed using the Randles circuit shown in the illustration in Figure S2 [22]. The calculated R_ct_ values of GCE, MOF-5, MWCNTs, and the MOF-5@MWCNTs composite are 838.8, 4810.2, 34.9, and 101.7 Ω., respectively. Compared with MOF-5, the in situ insertion of MWCNTs can effectively reduce the electron transfer resistance of MOF-5@MWCNTs composites. Furthermore, electrochemically active surface areas (EASA) of MOF-5@MWCNTs/GCE were evaluated using cyclic voltammetry at scan rates ranging from 0.01 to 0.1 V s^−1^ in 0.1 M KCl solution containing 5.0 mM Fe(CN)_6_^3−/4−^ (Figure S3a). Based on the Randles–Sevcik equation [23,24,25]:

(1) $$I_{\mathrm{pa}}=(2.691\times 10^{5}{)n}^{3/2}\nu^{1/2}D^{1/2}\mathrm{Ac}$$

In Equation (1), n is the number of electrons involved in the reaction, A is the EASA (cm^2^), c is the concentration of the analyte (mol cm^−3^), D is the diffusion coefficient (7.6 × 10^−6^ cm^2^ s^−1^), and ν is the scan rate (V s^−1^). Evidently, there is a good linear dependence between the square root of the scan rate (v^1/2^) and the anode peak currents (I_pa_) on MOF-5@MWCNTs/GCE (Figure S3b). Thereby, the EASAs of MOF-5@MWCNTs/GCE can been calculated to be 0.139 cm^2^ according to Equation (1), which is higher than that of MOF-5/GCE (0.014 cm^2^) (Figure S3c,d). This result means MOF-5@MWCNTs/GCE has more catalytic sites for the electrochemical analysis of AP and DA when the MWCNTs are introduced into MOF-5.

### 2.3. The Electrochemical Detection of AP and DA on MOF-5@MWCNTs

**Figure 4:**
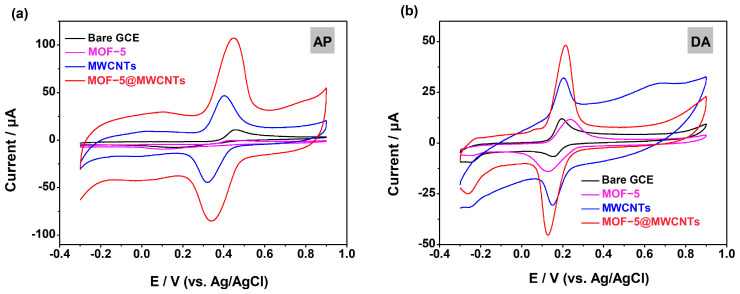
CVs of AP (**a**) in 0.1 M PBS (pH = 7) and DA (**b**) in 0.1 M PBS (pH = 6) on GCE, MOF-5/GCE, MWCNTs/GCE and MOF-5@MWCNT/GCE.

Cyclic voltammetry (CV) was used to detect the electrochemical response of AP and DA on different modified electrodes (Figure 4) in an electrolytic cell containing 10 mL of phosphate-buffer solution (PBS). The results show that AP only exhibits weak and irreversible redox peaks on the surface of the bare GCE. Compared with MOF-5/GCE and MWCNTs/GCE, MOF-5@MWCNTs/GCE showcases the maximum peak current and strongly symmetry peaks. Specifically, the oxidation peak current (I_pa_) of AP on the MOF-5@MWCNTs/GCE (107.16 μA) is about 2.29 times and 510 times higher than that on the MWCNTs/GCE (I_pa_ = 46.86 μA) and MOF-5/GCE (I_pa_ = 0.21 μA), respectively, proving that MOF-5@MWCNTs/GCE has the highest electrochemical activity during AP detection (Figure 4a). As for the detection of DA (Figure 4b), the weak current responses appeared on the bare GCE and MOF-5/GCE. After modification with MOF-5@MWCNTs, I_pa_ significantly increases up to 48.06 μA, which is 4.12 times and 1.50 times higher than that on MOF-5/GCE (11.66 μA) and MWCNTs (32.04 μA), respectively. 

Both AP and DA are detected, and MOF-5@MWCNT shows an excellent electrochemical response. Based on these experimental results, there should be a synergistic effect between MOF-5 and MWCNTs for the detection of AP and DA. Combined with results published in the literature, a reaction mechanism for the reactants and electrode materials is proposed below [26,27,28]:

(1) MWCNTs + AP(DA)→MWCNTs-AP(DA).
(2) MWCNTs-AP(DA) − e^−^→MWCNTs-Ox-AP(DA).
(3) MWCNTs-Ox-AP(DA) + MOF-5→MOF-5-Ox-AP(DA) + MWCNTs

Rate-determining step.

(4) MOF-5-Ox-AP(DA)→MOF-5 + Ox-AP(DA).

The electrochemical reaction process of AP or DA on the electrode surface could be described as follows: In step (1), AP or DA is adsorbed on the surface of MWCNTs. Then, the adsorbed AP or DA is oxidized to form MWCNTs-Oxd-AP or MWCNTs-Oxd-DA and loses electrons, which pass through the MWCNTs to the GCE (Step (2)). In step (3), Oxd-AP or Oxd-DA is transferred to the MOF-5 surface from MWCNTs to produce MOF-5-Oxd-AP or MOF-5-Oxd-DA. In step (4), Oxd-AP or Oxd-DA is dissociated from MOF-5 and diffuses into the solution. Because the conductivity of MOF-5 is poor, electron transfer should mainly occur among AP or DA molecules, MWCNTs, and GCE (Figure S4).

According to the proposed mechanism, the in situ synthesis of MOF-5 and MWCNTs may be very important for the synergistic effect [22]. As a comparison, a new modified electrode, MOF-5/MWCNTs/GCE, was prepared through a simple grinding method for MOF-5 and MWCNTs. In theory, the response current of MOF-5/MWCNTs/GCE for AP and DA would be lower than that of MOF-5@MWCNTs/GCE if the proposed mechanism is correct [22]. More importantly, the response currents are 39.45 μA for AP and 33.76 μA for DA on the MOF-5/MWCNTs/GCE, which are clearly lower than those on the MOF-5@MWCNTs/GCE (107.10 μA for AP and 48.06 μA for DA) (Figure S5). The experimental results are in good agreement with the theoretical prediction [22]. To further validate the proposed mechanism, additional carbon materials, including reduced graphene oxide (rGO) and mesoporous carbon (MC), were synthesized and recombined with MOF-5 via in situ synthesis. Given the presence of hydroxyl and carboxyl groups on rGO, it can interact with oxidized AP (or DA) through hydrogen bonding. Due to the many channels within MC, the departure of oxidized AP (or DA) from these channels is challenging [22,26]. Therefore, the bonding strength between MWCNTs and oxidized AP (or DA) is weaker compared to that between rGO (or MC) and oxidized AP (or DA). However, MOF-5@MWCNTs exhibits superior catalytic properties compared to MOF-5@rGO and MOF-5@MC (Figure S6). Therefore, the proposed mechanism is deemed accurate.

#### 2.3.1. Effect of pH on AP and DA Response on the MOF-5@MWCNTs/GCE

**Figure 5:**
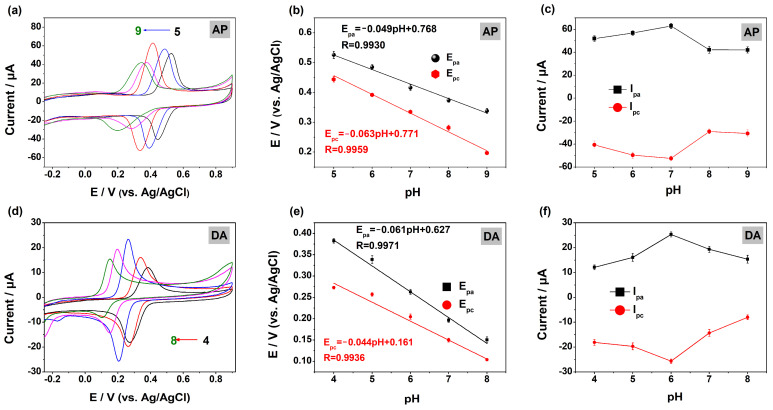
CVs of (**a**) AP and (**d**) DA in PBS (0.1 M) with different pH on MOF-5@MWCNTs/GCE. Effect of pH on E_p_ and I_p_ for AP (**b**,**c**) and DA (**e**,**f**).

The electrochemical response of AP in PBS at different pH levels was studied by CV. Figure 5a shows that the peak potentials (E_p_), including the oxidation peak (E_pa_) and the reduction peak potential (E_pc_) of AP, shift in a negative direction when the pH increases, indicating that protons are involved in the electrode reaction of AP [29]. And, the corresponding regression equations can be expressed as E_pa_(V) = −0.049 pH + 0.768 (R = 0.9930) and E_pc_(V) = −0.063 pH + 0.771 (R = 0.9959) (Figure 5b). Similarly, both E_pa_ and E_pc_ for DA also move in a negative direction and change linearly (Figure 5d), and their corresponding linear regression equations are E_pa_(V) = −0.061 pH + 0.627 (R = 0.9971) and E_pc_(V) = −0.044 pH + 0.161 (R = 0.9936) (Figure 5e). The slope values in the four equations approximate the theoretical value of -0.059, indicating that the number of protons involved in the electrocatalysis of AP (or DA) is equal to the number of electrons according to Nernst’s equation [30]. Additionally, it can be seen from Figure 5c that, in a PBS with pH = 7.0, the oxidation peak current (I_pa_) and reduction peak current (I_pc_) are the largest for AP, indicating that neutral test conditions are the most beneficial for MOF-5@MWCNTs to detect AP. For the subsequent electrochemical determination of DA, pH 6.0 was chosen (Figure 5f).

#### 2.3.2. Kinetic Study of AP and DA on the MOF-5@MWCNTs/GCE

**Figure 6:**
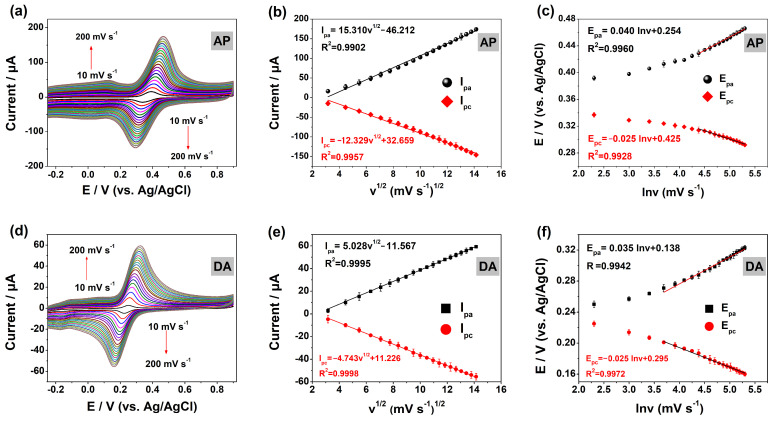
CVs of (**a**) AP and (**d**) DA in PBS (0.1 M) with different scan rates (10–200 mV s^−1^, different colored lines represent different scan speeds, and the CV curve is scanned for every 10 mV s^−1^ increases in scan speed) on MOF-5@MWCNTs/GCE. Effect of scan rates on I_p_ and E_p_ for AP (**b**,**c**) and DA (**e**,**f**).

**Figure 7:**
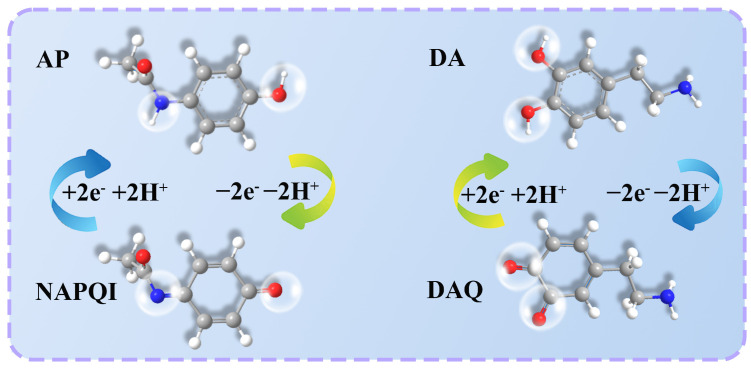
Mechanism governing the oxidation of AP and DA into NAPQI (N-Acetyl-P-Benzoquinone-imine) and DAQ (dopamine quinine).

The effect of the scanning rate on the redox peak current and peak potentials in the respective PBS with the optimum pH for AP and DA was investigated by CV. Figure 6a,d show the CV curves for a scan rate ranging from 10 to 200 mV/s. As the scan rate increases, the peak current (I_p_) becomes linearly correlated with the square root of the scan rate (v^1/2^). The correlative equations are I_pa_ (μA) = 15.310 v^1/2^ (m V/s)^1/2^ − 46.212 (R^2^ = 0.9902) and I_pc_ (μA) = −12.329 v^1/2^ (m V/s)^1/2^ + 32.659 (R^2^ = 0.9957) for AP (Figure 6b). Meanwhile, as shown in Figure 6e, for DA, the correlative equations are I_pa_ (μA) = 5.028 v^1/2^ (m V/s)^1/2^ − 11.567 (R^2^ = 0.9995) and I_pc_ (μA)= −4.743 v^1/2^ (m V/s)^1/2^ + 11.226 (R^2^ = 0.9998). These results mean that the electrocatalytic behavior of AP or DA on the MOF-5@MWCNTs/GCE is primarily a diffusion-controlled process [31]. Additionally, as the scan rate is augmented, the E_pa_ shifts towards the positive direction, while the E_pc_ shifts towards the negative direction (Figure 6a,d). And, the linear relationships between the peak potentials and logarithms of the scan rates (lnv) are E_pa_ = 0.040lnν + 0.254 (R^2^ = 0.9960) and E_pc_ = −0.025lnν + 0.425 (R^2^ = 0.9928) for AP (Figure 6c). Similarly, the linear relationships for DA can been described as E_pa_ = 0.035lnν + 0.138 (R = 0.9942) and E_pc_ = −0.025lnν + 0.295 (R^2^ = 0.9972) (Figure 6f). Using the Laviron Equations (2) and (3) [22], the transfer numbers (n) of electrons for AP and DA were calculated to be 1.69 and 1.74, respectively. This indicates that the electrochemical reaction of AP or DA on MOF-5@MWCNTs/GCE is a two-proton, two-electron transfer process. Thus, the possible reaction process of AP and DA on the MOF-5@MWCNTs/GCE can be represented by Figure 7.

(2) $$E_{\mathrm{pa}}=E^{\theta }+\frac{\mathrm{RT}}{\left(1-\alpha \right)\mathrm{nF}}\ln\nu$$

(3) $$E_{\mathrm{pc}}=E^{\theta }-\frac{\mathrm{RT}}{\alpha \mathrm{nF}}\ln\nu$$

#### 2.3.3. Quantitative Detection of AP and DA on MOF-5@MWCNTs/GCE

**Figure 8:**
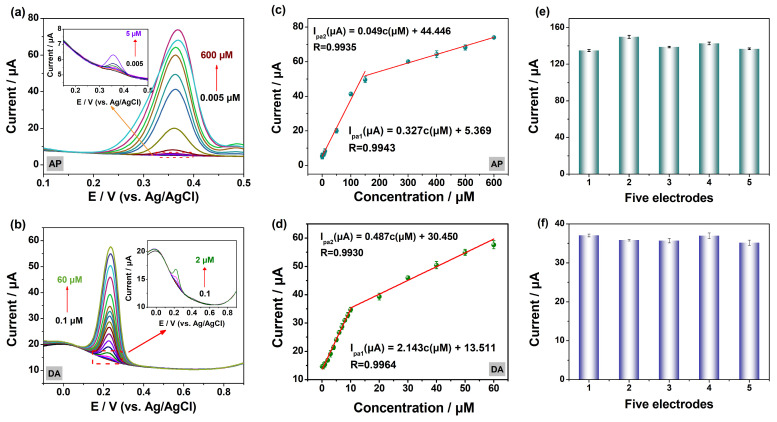
DPV curves of AP (0.005–600 μM) (**a**) and DA (0.1–60 μM) (**b**) on MOF-5@MWCNTs/GCE. Linear curve for AP (**c**) and DA (**d**) concentrations of versus peak currents (*n* = 3, which is a multiple of noise). (**e**,**f**) Current responses of five parallel MOF-5@MWCNTs/GCE for the detection of AP and DA.

A quantitative analysis of AP and DA was carried out via differential pulse voltammetry (DPV), a high-sensitivity electrochemical analysis method, under the optimum conditions. As shown in Figure 8a,b, the DPV response signal gradually enhances with the increase in AP or DA concentration. For AP, the I_pa_ on MOF-5@MWCNTs/GCE shows a linear increase with increasing AP concentration (0.005–600 μM, Figure 8a). The corresponding linear equations are I_pa1_ (μA) = 0.327c (μM) + 5.369 (R = 0.9943) for low concentrations (0.005–150 μM) and I_pa2_ (μA) = 0.049c (μM) + 44.446 (R = 0.9935) for high concentrations (150–600 μM) (Figure 8c). From the linear equations, it can be calculated that the detection limit (LOD) of AP on the MOF-5@MWCNTs/GCE is 0.061 μM (S/N = 3). As shown in Figure 8d, there are two distinct linear relationships between the concentration of DA and the peak current as the concentration of DA increases from 0.1 to 60 μM. The first linear relationship can be expressed as I_pa1_ (μA) = 2.143c (μM) + 13.511 (R = 0.9964) for concentrations ranging from 0.1 to 10 μM, while the second linear relationship can be expressed as I_pa2_ (μA) = 0.487c (μM) + 30.450 (R = 0.9930) for concentrations ranging from 10 to 60 μM. And the LOD of DA is 0.0075 μM on this electrochemical sensor based on MOF-5@MWCNTs. In order to reasonably evaluate the performance of the electrochemical sensor based on MOF-5@MWCNT in the detection of AP and DA, Table 1 compares the detection performance of this sensor with related work published in recent years. As can be seen from Table 1 [32,33,34,35,36,37,38,39,40,41,42,43,44,45,46,47,48,49,50,51,52,53,54,55,56,57], some of the reported sensors do not have the dual function of detecting AP and DA, while this sensor based on the MOF-5@MWCNT can offer an expanded linear response span and diminished limits of detection for both analytes, revealing that the proposed sensors show great advantages in the detection of AP and DA.

#### 2.3.4. Reproducibility, Stability, and Anti-Interference

The sensor must have good reproducibility, stability, and anti-interference abilities to have practical application value. Therefore, this experiment conducted research and testing on the reproducibility, stability, and anti-interference of the electrochemical sensor based on MOF-5@MWCNTs. Firstly, five identical working electrodes modified by MOF-5@MWCNTs were prepared in parallel to determine the levels of AP or DA through DPV, and the relative deviation (RSD) of the current response from the five electrodes was 4.22% for AP (Figure 8e) and 2.27% for DA (Figure 8f), indicating that this electrochemical sensor shows good reproducibility in detecting AP or DA [37]. Furthermore, the DPV method was used to investigate the stability of the sensor for determining AP and DA. The GCE modified with MOF-5@MWCNTs was stored at 4 °C, and the current response of AP or DA was recorded every 48 h. The experimental results show that the current response of AP and DA still maintains approximately 92.2% and 90.4% of the initial current value after 9 days, respectively (Figure S7), which indicates that the sensor based on MOF-5@MWCNTs/GCE possesses a good stability and also means that the electrode can maintain a life of at least 9 days in the conditions under which AP and DA were tested [43]. Finally, anti-interference experiments were performed to evaluate the selectivity of this sensor by adding high concentrations of various interferents to the PBS containing AP or DA, including 10-fold concentrations of inorganic ions (Na^+^, K^+^, Mg^2+^, SO_4_^2−^, Cl^−^) and 2-fold concentrations of ascorbic acid (AA) and glucose, as well as equal concentrations of chloramphenicol and penicillin. Clearly, the MOF-5@MWCNTs/GCE sensor can still be used to detect AP and DA in the presence of these interfering compounds (Figure S8). In summary, it can be concluded that the designed electrochemical sensor has a good anti-interference ability [57].

#### 2.3.5. Analysis for Real Samples

**Figure 9:**
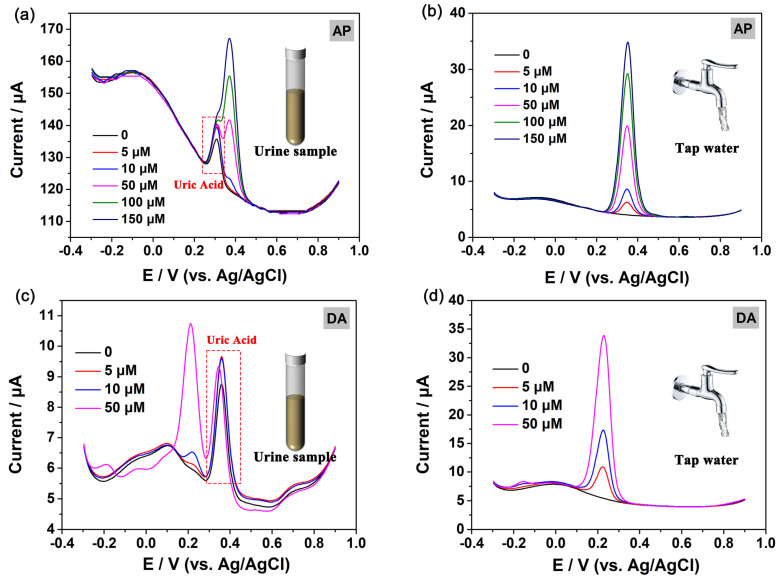
DPV responses of the added AP standard solution (5, 10, 50, 100 and 150 μM) in the urine (**a**) and tap water (**b**) samples and DA standard solution (5, 10, 50 μM) in the urine (**c**) and tap water (**d**) samples.

To further validate the efficacy of the MOF-5@MWCNTs/GCE sensor in real-world applications, DPV was employed to quantify the amount of AP and DA in tap water and urine using the standard addition method. The tap water and urine were collected and diluted ten and twenty times using PBS, respectively, without any further pretreatment. As shown in Figure 9, the assay reveals recovery rates of 98.2% to 104.2% for AP and 97.6% to 104.4% for DA (summarized in Table S1), accompanied by relative standard deviations (RSDs) below 5% [56]. These findings robustly confirm the sensor’s capability to accurately determine AP and DA concentrations in authentic samples, thereby endorsing its practical utility for the analysis of real samples.

## 3. Experimental Section

### 3.1. Materials and Reagents

Zinc acetate dihydrate ((CH_3_COO)_2_Zn·2H_2_O, 99%) was purchased from Tianjin Guangfu Technology Development Co., Ltd., Tianjin, China, Terephthalic acid (BDC, 98%), multi-walled carbon nanotubes (MWCNTs), acetylaminophenol (AP, C_8_H_9_NO_2_, 99%), and dopamine (DA, 98%) were bought from Shanghai Maclin Biochemical Technology Co., LTD., Shanghai, China. Anhydrous ethanol (CH_3_CH_2_OH, 99.7%), N, N-dimethylformamide (DMF, 99.5%) and dichloromethane (CH_2_Cl_2_, 99.5%) were purchased from Tianjin Yongda Chemical reagent Co., Ltd., Tianjin, China.

### 3.2. Synthesis of MOF-5

Zinc acetate dihydrate (0.6146 g, 2.80 mmol) and terephthalic acid (0.1756 g, 1.06 mmol) were dissolved in 20 mL of N, N-dimethylformamide (DMF) and ultrasonicated at room temperature for 60 min. The obtained suspension was transferred to a reaction vessel equipped with a polytetrafluoroethylene (PTFE) liner. It was then placed in a vacuum oven and reacted for 14 h at a temperature of 130 °C. After cooling at room temperature, the product was washed three times with DMF and anhydrous ethanol, respectively, and then dried at 70 °C for 1 h. Finally, the obtained white powder was soaked in a certain amount of dichloromethane for 8 h to remove residual DMF, before being washed three times with anhydrous ethanol and dried at 70 °C for 2 h.

### 3.3. Preparation of MOF-5@MWCNTs

The MOF-5@MWCNTs composite material was prepared using the same method used to synthesize MOF-5. The difference lies in the addition of 0.05 g of MWCNTs powders, which was dispersed into the above suspension and ultrasonicated for 60 min.

### 3.4. Preparation of Electrochemical Sensors

To prepare the electrode for modification, a glassy carbon electrode (GCE) was polished with 1.0, 0.3, and 0.05 μm Al_2_O_3_ polishing powder until the electrode surface was smooth and mirror-like. The electrode was then cleaned alternately with ultrasonic treatment using ethanol and deionized water and dried at room temperature. A dispersion solution was prepared by mixing 5 mg of MOF-5@MWCNTs powder with 1 mL of a 0.5 wt% Nafion solution under ultrasonic action for 1 h. Then, 5 μL of the MOF-5@MWCNTs dispersion was deposited onto the surface of the GCE and dried at room temperature. For comparison, dispersion of MOF-5 or MWCNTs alone was also applied to the GCE in the same manner. The electrochemical properties of the MOF-5, MWCNTs, and MOF-5@MWCNTs catalysts prepared on the three electrodes were studied using an electrochemical workstation (CHI760e). The three-electrode system consisted of a saturated Ag/AgCl electrode as the reference electrode, a Pt plate as the counter electrode, the GCE modified by the catalyst as the working electrode, and phosphate-buffer solution (PBS) as the electrolyte solution.

## 4. Conclusions

A low-cost, efficient, and dual-functional MOF-5@MWCNTs composite was successfully synthesized using a simple solvothermal method. And the electrochemical sensor based on this composite can achieve the trace detection of AP and DA, characterized by very low limits of detection, expansive linear ranges, outstanding reproducibility, stability, and a robust resistance to interference. Significantly, the practical application of this sensor in the analysis of AP and DA within real samples, specifically tap water and urine, yielded satisfactory outcomes. This study thus establishes a potent platform for the quantitative electrochemical detection of drugs and living molecules. Looking ahead, efforts are directed towards refining the detection limits of MOF-5@MWCNTs to cater to more stringent analytical demands. In future sensor designs, the focus will lie on exploring metal–organic frameworks with heightened affinity for target analytes, aimed at bolstering the electrochemical sensing performance. Moreover, optimizing the synergistic interaction between the metal–organic framework and the conductive substrate represents a promising strategy to further enhance the sensor’s electrochemical characteristics, thereby pushing the boundaries of analytical sensitivity and precision.

## Figures and Tables

**Table 1:** Comparison of different modifiers used in AP and DA measurement.

Electrode Material	Linear Range (µM)		LOD (µM)		Refs.
	AP	DA	AP	DA	
C-ZIF-67/SP/GCE	0.5–100	1–100	0.27	0.58	[32]
3D RGO/MWCNTs@ZrFeO_x_	1–14 14–190	1–12, 12–180	0.230	0.212	[29]
UiO-66-NH_2_/CNTs/GCE	0.03–2	0.03–2	0.009	0.015	[33]
Ce-MOF-COOH/CB_0.4_	2.5–617	2.5–310	0.057	0.03	[34]
CeO_2_:BaMoO_4_/3DPE electrode	-	5–1000 μM	-	2.77	[35]
The clavate-shaped BiVO_4_	0.5–100	-	0.2	-	[36]
Co, Mo@CNFs	-	0.01–1000	-	0.00235	[37]
Co_2_P hybrid	-	0.2–10, 10–50	-	0.018	[38]
CoFe-LDH/GCE	2–900	2–900	0.37	0.08	[39]
CuNi-MOF@rGO	-	1–500	-	9.41	[40]
CuO-Cu_2_O	5–100	2–100	0.06	0.1	[41]
CoP_3_/Cu_3_P NRs/CF	-	0.2–2000	-	0.51	[42]
Fcmc/Ni-Pd/f-MWCNT	-	2–400	-	0.05	[43]
FeCo@C	0.001–0.8	-	0.59	-	[44]
FeS_2_/CoS_2_NHs/GCE	1–27,000	1–24,000	0.8	1.1	[45]
MIL-125(Ti–Al)-75%NH_2_	-	5–100	-	0.0876	[46]
MoS_2_/rGO/GCE	-	0.1–110.0	-	0.013	[47]
N-HKUST-1/Au composite	1–4448.4	-	0.16	-	[48]
Ni-MOFs/MWCNTs	-	2–200	-	0.017	[49]
NiO–ZnO/rGO	-	0.0041–0.054	-	0.0076	[50]
PS-PDEA-PS/MWCNT–COOH	1.5–85.1 85.1–235.1	-	0.57	-	[51]
Pt/Ppy	4–150	0.1–8	0.9	0.078	[52]
Ti_3_C_2_/CoNP-NCNTHP	0.5–350	-	0.05	-	[53]
Ti_3_C_2_T_x_@TiO_2_ NSs	-	40–300	-	0.19	[54]
TM-CNT600	-	10.7–24.2	-	1.42	[55]
ZnCo_2_O_4_@Fe_3_O_4_/MWCNTs	1.0–150.0	-	0.018	-	[1]
Zn-rich Zn-Co BTC MOF	-	0.1–500	-	0.04	[56]
WBiVO_4_/f-MWCNTs/SPCE	0.5–36.5 48.5–158.5	-	0.082	-	[3]
CoNi-MOF@ERGO/GCE	-	0.1−400	-	0.086	[57]
MOF-5@MWCNTs	0.005–150, 150–600	0.1–10, 10–60	0.061	0.0075	This work

## Data Availability

Data are contained within the article.

## Supplementary Materials

The following supporting information can be downloaded at: https://www.mdpi.com/article/10.3390/molecules29235534/s1, Figure S1. N_2_ adsorption–desorption isotherms (a) and corresponding pore size distribution curves (b) of MOF-5, MWCNTs and MOF-5@MWCNTs; Figure S2. EIS of MOF-5, MWCNTs and MOF-5@MWCNTs recorded in 5.0 mM Fe(CN)_6_^3−/4−^ with 0.1 M KCl, thefrequency range from 0.1 Hz to 10.0 kHz (Inset: the Randles circuit); Figure S3. The CV curves under different scan rates (10 mV/s to 100 mV/s) for MOF-5 (a), MOF-5@MWCNTs (c) in 5.0 mM Fe(CN)_6_^3−/4−^ with 0.1 M KCl. (b,d) Corresponding linear plots of Ipa versus v^1/2^; Figure S4. Take AP as an example, the electrocatalytic process of MOF-5@MWCNTs towards AP (The picture on the right is a real view of the electrode reaction in the left); Figure S5. CV curves of AP (a) and DA (b) on MOF-5@MWCNTs (in situ synthesis) and MOF-5/MWCNTs (simple grinding) modified GCE; Figure S6. CV curves of AP (a) and DA (b) on MOF-5@MWCNTs, MOF-5@rGO and MOF-5@MC modified GCE in the same conditions; Figure S7. Stability study of MOF-5@MWCNTs/GCE for the determination of AP (a) and DA (b); Figure S8. The anti-interference ability evaluation of electrochemical sensor based on the MOF-5@MWCNTs for the detection of AP (a) and DA (b); Table S1 Determination of DA and AP in actual samples by electrochemical sensor based on MOF-5@MWCNTs.
