# Supplementary material for: A Dual-Functional and Efficient MOF-5@MWCNTs Electrochemical Sensing Device for the Measurement of Trace-Level Acetaminophenol and Dopamine
Source: Molecules. 2024 Nov 23;29(23):5534. doi: 10.3390/molecules29235534 (PMC11643747; doi:10.3390/molecules29235534)
Supplement: Supplementary file 1 [file molecules-29-05534-s001.zip › molecules-3305885-supplementary.pdf]

A Dual-Functional and Efficient MOF-5@MWCNTs Electrochemical Sensing Device for the Measurement of Trace-Level Acetaminophenol and Dopamine

Jianxia Gu *, Shuting Lang, Zhanbin Jin and Tingting Wei

Department of Chemistry, Xinzhou Normal University, Xinzhou 034000, China

* Correspondence: gujx349@xztu.edu.cn

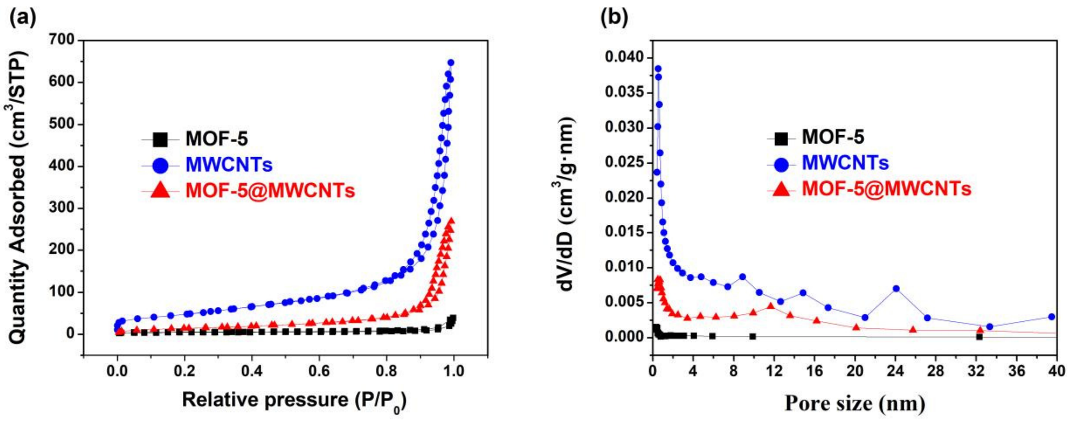

Figure S1. N_2 adsorption-desorption isotherms (a) and corresponding pore size distribution curves (b) of MOF-5, MWCNTs and MOF-5@MWCNTs.

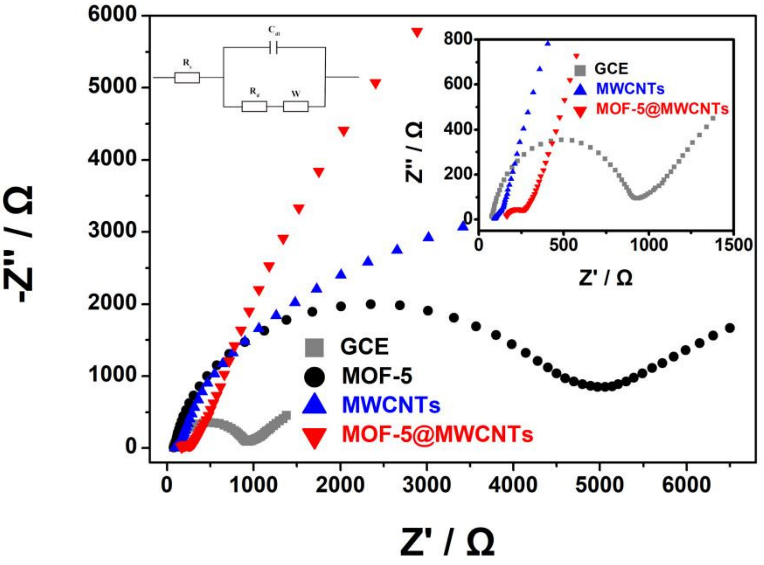

Figure S2. EIS of MOF-5, MWCNTs and MOF-5@MWCNTs recorded in 5.0 mM $Fe(CN)_6^{3-/4-}$ with 0.1 M KCl, thefrequency range from 0.1 Hz to 10.0 kHz (Inset: the Randles circuit)..

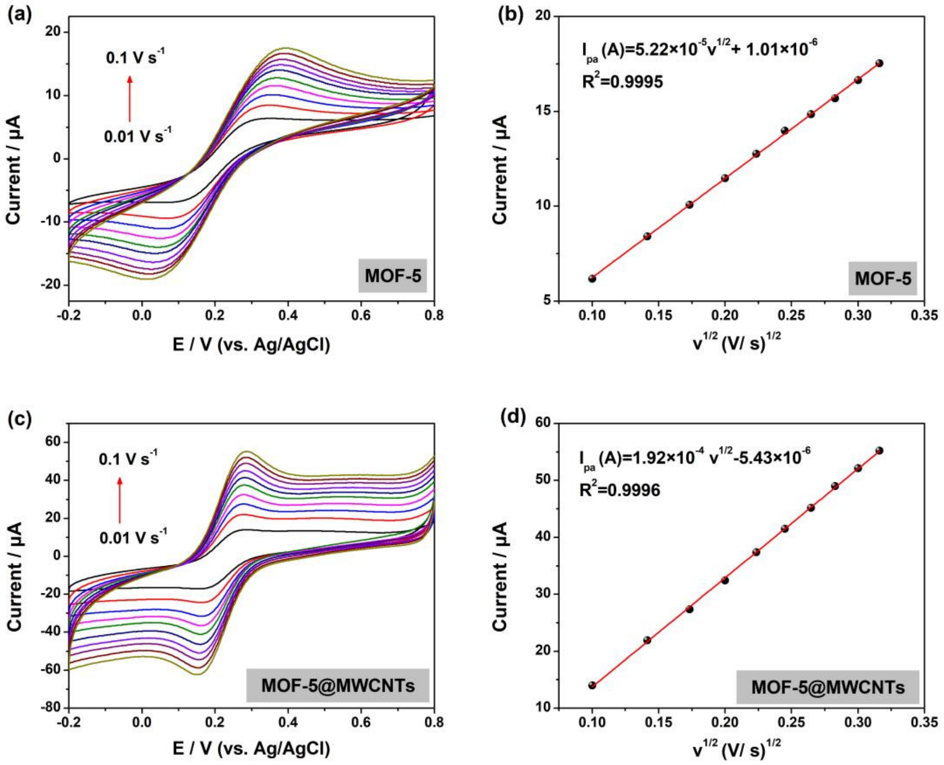

Figure S3. The CV curves under different scan rates (10 mV/s to 100 mV/s) for MOF-5 (a), MOF-5@MWCNTs (c) in 5.0 mM $Fe(CN)_6^{3-/4-}$ with 0.1 M KCl. (b, d) Corresponding linear plots of Ipa versus $v^{1/2}$.

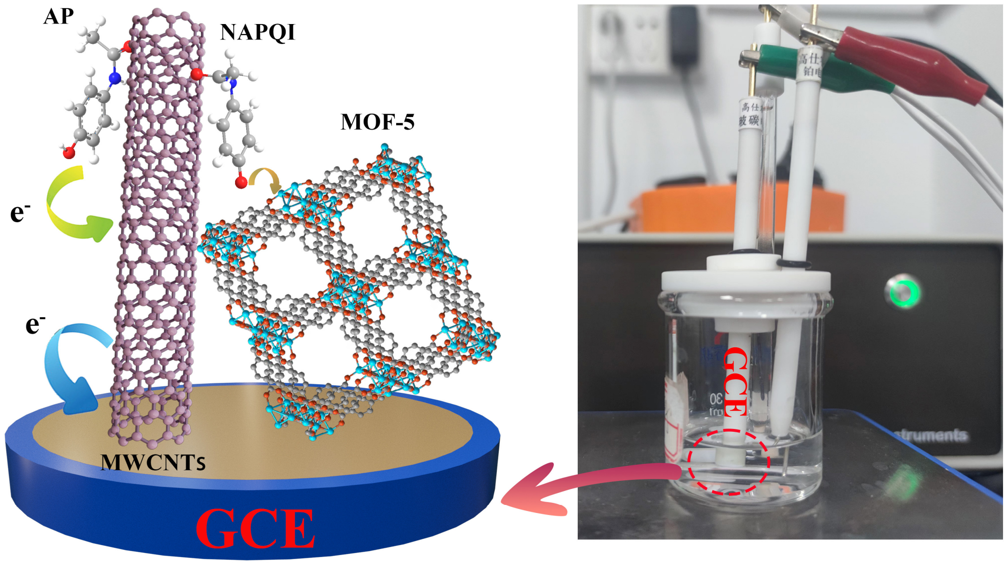

Figure S4. Take AP as an example, the electrocatalytic process of MOF-5@MWCNTs towards AP (The picture on the right is a real view of the electrode reaction in the left).

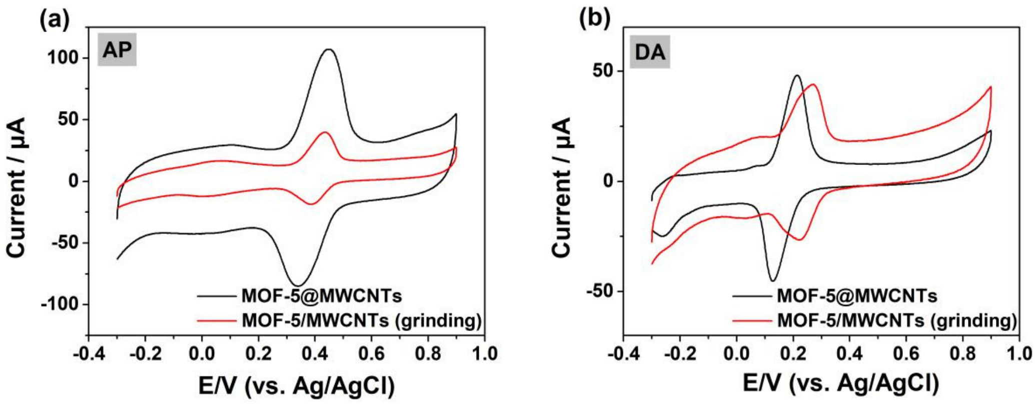

Figure S5. CV curves of AP (a) and DA (b) on MOF-5@MWCNTs (in situ synthesis) and MOF-5/MWCNTs (simple grinding) modified GCE.

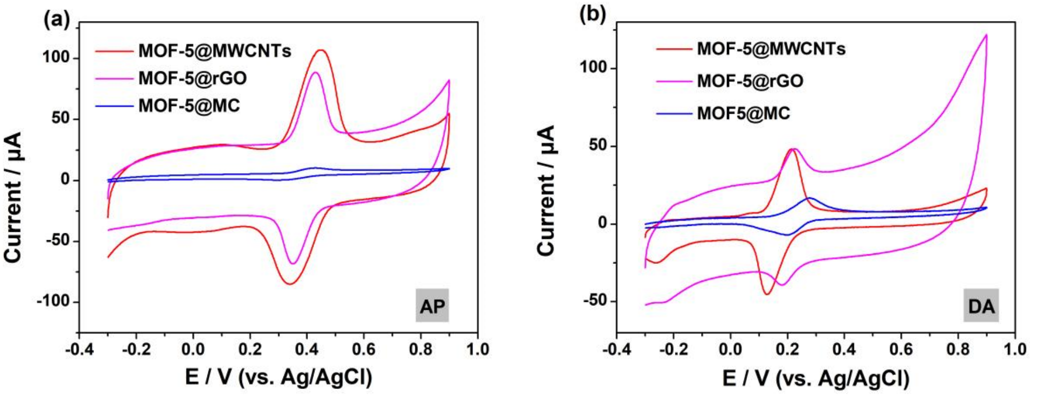

Figure S6. CV curves of AP (a) and DA (b) on MOF-5@MWCNTs, MOF-5@rGO and MOF-5@MC modified GCE in the same conditions.

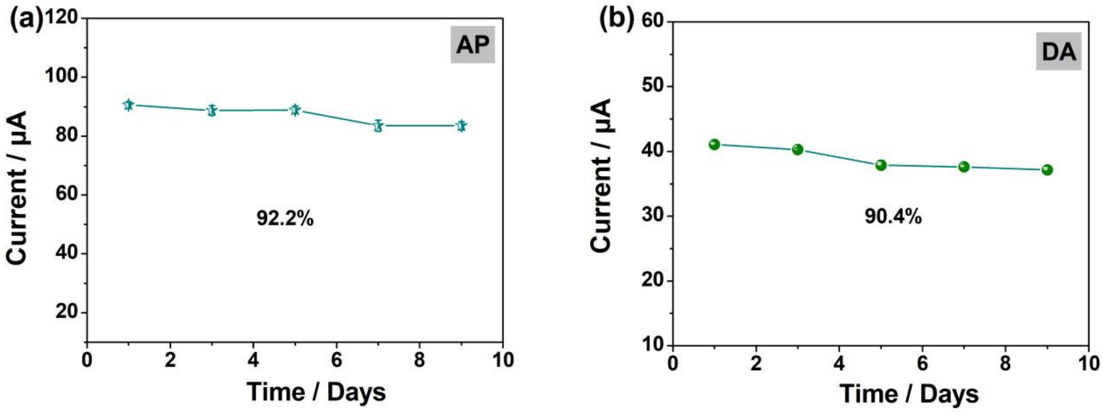

Figure S7. Stability study of MOF-5@MWCNTs/GCE for the determination of AP (a) and DA (b).

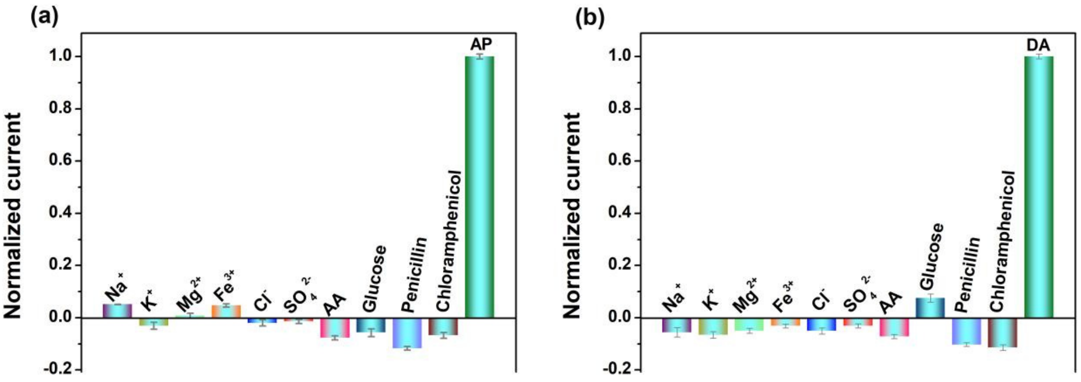

Figure S8. The anti-interference ability evaluation of electrochemical sensor based on

the MOF-5@MWCNTs for the detection of AP (a) and DA (b).

Table S1 Determination of DA and AP in actual samples by electrochemical sensor based on MOF-5@MWCNTs.

Samples	Added (μM)		Recovered (μM)		Recovery (%)		RSD (%)	
	AP	DA	AP	DA	AP	DA	AP	DA
Tap Water	50	50	49.19	48.81	98.4	97.6	3.0	3.6
	50	50	49.88	52.18	99.8	104.4		
	50	50	52.12	49.34	104.2	98.7		
Urine	50	50	52.42	51.65	104.8	103.3	3.3	0.4
	50	50	49.09	52.10	98.2	104.2		
	50	50	50.30	51.96	100.6	103.9		
